# Phenolic Profiles and Antioxidant Properties of Young Wines Made from Yan73 (*Vitis vinifera* L.) and Cabernet Sauvignon (*Vitis vinifera* L.) Grapes Treated by 24-Epibrassinolide

**DOI:** 10.3390/molecules190710189

**Published:** 2014-07-14

**Authors:** Fan Xu, Li-Ying Luan, Zhen-Wen Zhang, Shan-Shan Huo, Xiang Gao, Yu-Lin Fang, Zhu-Mei Xi

**Affiliations:** 1 College of Enology, Northwest A&F University, Yangling 712100, China; E-Mails: xufanwine@126.com (F.X.); luanliying@163.com (L.-Y.L.); zhangzhw60@nwsuaf.edu.cn (Z.-W.Z.); huoshanshan68@163.com (S.-S.H.); present816@126.com (X.G.); fangyulin@nwsuaf.edu.cn (Y.-L.F.); 2 Shaanxi Engineering Research Center for Viti-Viniculture, Yangling 712100, Shaanxi, China

**Keywords:** phenolic profiles, antioxidant capacity, Yan73, Cabernet Sauvignon, wine, 24-epibrassinolide

## Abstract

The grape berries of two varieties, Yan73 (*Vitis vinifera* L*.*) and Cabernet Sauvignon (CS) (*Vitis vinifera* L*.*) were treated with 0.40 mg/L 24-epibrassinolide (EBR), 1.00 mg/L brassinazole (Brz), and deionized water (control), at the veraison period. The EBR treatment significantly increased total phenolic content (TPC), total tannin content (TTC) and total anthocyanin content (TAC) of Yan73 and CS wines, whereas Brz treatment decreased TPC, total flavonoid content (TFC), TAC in the two wines. Moreover, the content of most of the phenolic compounds identified by HPLC-DAD/ESI-MS in EBR-treated wines was significantly higher than that in control. The antioxidant capacities, which determined using DPPH, ABTS and HRSA methods, of the wines were increased by EBR treatment as well. There was a good correlation between the antioxidant capacity and phenolic content. The results demonstrated that EBR could enhance the phenolic compounds and antioxidant capacity of Yan73 and CS wines, but the effects may vary by different cultivars.

## 1. Introduction

Phenolic compounds represent some of the most important constituents affecting the quality parameters of wines due to their direct influence on some important organoleptic characteristics of wines, such as color, flavor, bitterness and astringency [1]. Common phenolic compounds in wines, such as anthocyanins, flavanols and tannins, have been shown to be good source of phenolic antioxidants. The antioxidative properties of these compounds may protect against arteriosclerosis and coronary heart disease [2,3], therefore, the phenolic composition of wines has been studied by many researchers [4,5,6,7]. The cultivation method to promote phenolic composition in wines is one of the current research focuses. Within the same grape variety, the growing season, environmental and climatic conditions, plant disease, soil type, geographic locations, and maturity could influence the concentration of phenolic compounds [8,9]. Also, the plant hormones could affect the synthesis of these compounds. Some plant hormones, such as abscisic acid (ABA) [10,11], and brassinosteroids (BRs) [12,13] are involved in the increase of phenolic compounds in grape skins. Several studies have revealed that exogenous ABA application stimulates the biosynthesis of phenylpropanoids and flavonols in grape skins with an enhanced expression of anthocyanin biosynthesis pathway genes, which increased phenolic compounds [4] and anthocyanins [14,15]. ABA treatment to increase phenolic compounds and antioxidant capacity of wine [10] has been reported as well. BRs are a group of steroidal plant hormones that are essential for normal plant development, including cell elongation, cell division, pollen tube growth, ethylene biosynthesis, reproductive development, as well as stress response [16,17]. Recent evidence suggested that BRs are the latest plant hormones involved in the ripening of grapes [12]. The expression analysis of the genes encoding BR biosynthesis enzymes and receptors during grape berry development revealed that the transcript accumulation patterns were consistent with a dramatic increase in endogenous BR levels applied at the onset of fruit ripening [12]. Contrary to exogenous EBR, brassinazole (Brz) delayed Cabernet Sauvignon grape [12] and Akihime strawberry fruit ripening [18] and prevented the ovaries of cucumber (*Cucumis sativus*) cultivars from growth [19]. Brz, a BRs biosynthesis inhibitor, is synthesized based on the structure of uniconazole and paclobutrazol and has a tertiary hydroxy group on the carbon adjacent to the carbon where a triazole ring is attached [20,21]. It induces morphological changes in plants by interfering with the biosynthesis of BRs via blocking the steps between campestanol and 6-deoxoteasterone and between 6-oxocampestanol and teasterone [20,21]. Extensive research over the past two decades has revealed the importance of BR in numerous processes [22].

In viticulture, Xi *et al.* [13] found that exogenous EBR treatment can significantly promote grape ripening and enhance phenolic contents and antioxidant capacity in grape skins. However, information of the effect of EBR treatment to wine grape berries on the phenolic profiles and antioxidant capacity of their red wines is limited. Two representative varieties grapes, Yan73 and Cabernet Sauvignon (CS), have been used in the present study. From a practical standpoint, the results should provide useful information for application and further research on BR to enhance phenolic compounds in grapes and increase the quality of wine.

## 2. Results and Discussion

### 2.1. Composition of the Wines

In this study, the alcoholicity, reducing sugar, volatile acid, total acidity and dry extract of wines were in the range of the maximum acceptable limits of OIV [23] (Table 1). EBR treatment significantly increased the alcoholicity and dry extract of both Yan73 and Cabernet Sauvignon (CS) wines and decreased the total acidity of CS wine compared with the control. However, both EBR and Brz treatments had no significant effect on the total acidity of Yan73 wine. Brz significantly decreased the alcohol content of Yan73 and CS wines.

**Table 1:** Physicochemical parameters of Yan 73 and Cabernet Sauvignon wines (n = 3).

Variety	Treatment	Alcoholicity (v/v, %)	Reducing sugar (g/L)	Volatile acid ^(i)^ (mg/L)	Total acidity ^(j)^ (g/L)	Dry extract (g/L)
Cabernet Sauvignon	EBR	10.46 ± 0.06 ^a^	1.80 ± 0.05 ^c^	0.33 ± 0.02 ^a^	7.00 ± 0.09 ^c^	24.00 ± 0.05 ^a^
	Brz	9.55 ± 0.11 ^c^	2.32 ± 0.03 ^a^	0.29 ± 0.02 ^a^	8.38 ± 0.10 ^a^	23.84 ± 0.06 ^b^
	Control	10.03 ± 0.08 ^b^	1.96 ± 0.07 ^b^	0.32 ± 0.02 ^a^	7.69 ± 0.20 ^b^	21.47 ± 0.08 ^c^
Yan 73	EBR	10.25 ± 0.25 ^a^	3.40 ± 0.15 ^b^	0.33 ± 0.03 ^a^	9.58 ± 0.22 ^a^	30.20 ± 0.15 ^a^
	Brz	9.50 ± 0.08 ^c^	3.96 ± 0.09 ^a^	0.31 ± 0.03 ^a^	9.90 ± 0.40 ^a^	29.63 ± 0.09 ^b^
	Control	9.90 ± 0.32 ^b^	3.66 ± 0.38 ^a, b^	0.34 ± 0.02 ^a^	9.80 ± 0.28 ^a^	27.34 ± 0.38 ^c^

Results are mean ± standard deviation. Different letters (a,b,c) within the same row indicate significant difference at *p* < 0.05 by Duncan’s test. EBR: 0.40 mg/L 24-epibrassinolide. Brz: 1.00 mg/L brassinazole. Control: water. ^(i)^ as acetic acid; ^(j)^ as H_2_SO_4_.

### 2.2. Total Phenolic, Tannin, Flavonoid and Anthocyanin Content in the Wines

**Figure 1:**
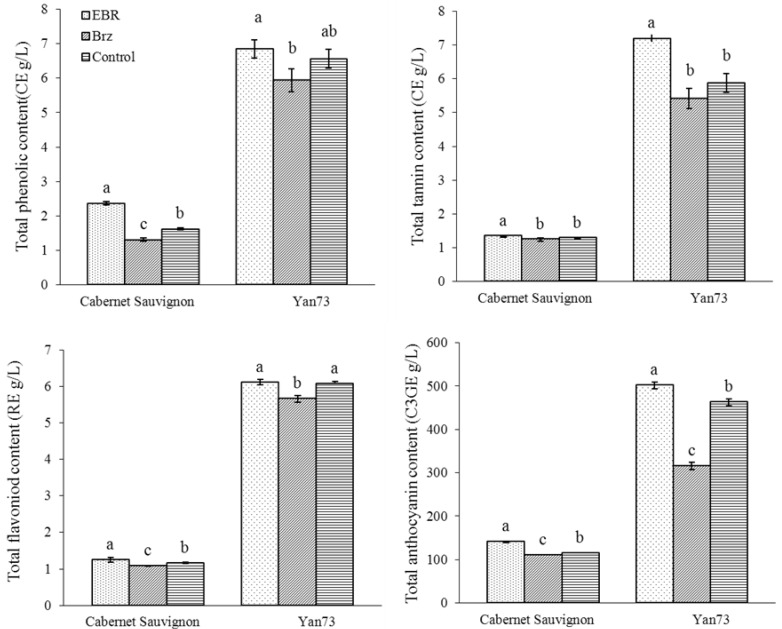
The effect of 24-epibrassinolide (EBR) and brassinazole (Brz) application to Yan73 and Cabernet Sauvignon on total phenolic, tannin, flavonoid, and anthocyanin contents of their wines (n = 3). Note: EBR: 0.40 mg/L 24-epibrassinolide. Brz: 1.00 mg/L brassinazole. Control: water. CE mg/L, RE mg/L, and C3GE mg/L represent milligrams of (+)-catechin equivalent, milligrams of rutin equivalent, and milligrams of cyanidin 3-glucoside equivalent per liter of wine, respectively. Values are mean ± SD values of 3 replicates. Different letters (a–c) within the same column indicate significant difference at *p* < 0.05 by Duncan’s test.

It was found that EBR or Brz treatments increased or decreased the TPC, TTC, TFC, TAC of Yan73 and CS wines, respectively (Figure 1). Among them, EBR increased significantly the TTC, and TAC of Yan73 wine and the TPC, TTC, TFC and TAC of CS wine. However, no significant difference in the TPC or TFC of Yan73 wine between the EBR treatment and control were observed. Opposite to EBR, Brz decreased significantly the TPC, TFC and TAC of CS wine and TFC and TAC of Yan73 wine, respectively. The TTC, TFC, or TAC of Yan73 wine and TPC, TFC, or TAC of CS wine for Brz treatment were significantly lower than for control. The decrease of TPC of Yan73 wine and TTC of CS wine was not significantly different from the control. Moreover, each of phenolic compounds in Yan73 wine was higher than that in CS wine.

### 2.3. Phenolic Profiles

For a better characterization of the phenolic composition of two cultivar wines, a detailed study by HPLC-DAD/ESI-MS was performed. Six classes of individual phenolic compounds were investigated and analyzed: anthocyanins, flavonols, flavan-3-ols, hydroxybenzoic acids, hydroxycinnamic acids and stilbenes. Fifty-two phenolic compounds were identified for most wines (Table 2).

#### 2.3.1. Anthocyanin Profiles

In this study, 21 individual anthocyanins were identified in the Yan73 wine, while 14 individual anthocyanins were found in the CS wine. The total content of anthocyains of Yan73 wine was 8.86 times higher than CS wine. The influence of EBR and Brz treatment on individual anthocyanin is reported in Table 2. All of the anthocyanins in Yan73 wine increased significantly by EBR treatment. The significant increase in total content of anthocyanins of Yan73 wine treated by EBR was observed

**Table 2:** Phenolic profiles of Yan73 and Cabernet Sauvignon wines (n = 3) produced with EBR-treated, Brz-treated, and untreated grape.

No.	Phenolic Compound	Cabernet Sauvignon			Yan 73			MS; MS^2^ (*m/z*)
		EBR	Brz	Control	EBR	Brz	Control	
Anthocyanins (mg ME/L)								
1	Cyanidin-3-*O*-glucoside	1.78 ± 0.07 ^a^	1.45 ± 0.01 ^b^	1.35 ± 0.08 ^c^	11.67 ± 1.70 ^a^	7.24 ± 0.26 ^b^	6.45 ± 0.37 ^b^	449(287)
2	Cyanidin-3-*O*-(6-*O*-acetyl)-glucoside	1.12 ± 0.03 ^a^	0.90 ± 0.01 ^b^	1.09 ± 0.01 ^a^	19.98 ± 5.23 ^a^	14.00±0.21 ^b^	9.00 ±0.02 ^c^	653 (611, 449, 287)
3	Cyanidin-3-*O*-(6-*O*-coumaryl)-glucoside	nd	nd	nd	5.87 ± 1.00 ^a^	1.14 ± 0.63 ^b^	1.48 ± 0.12 ^b^	595(287)
4	Delphinidin-3-*O*-glucoside	3.23 ± 0.02 ^a^	3.02 ± 0.07 ^b^	2.27±0.12 ^c^	171.06 ± 6.05 ^a^	31.89 ± 0.94 ^b^	26.27 ± 0.38 ^c^	465(303)
5	Dephinidin-3-*O*-(6-*O*-acetyl)-glucoside	2.01 ± 0.12 ^a, b^	1.93 ± 0.06 ^b^	2.12 ± 0.18 ^a^	67.14 ± 1.26 ^a^	43.29 ± 0.77 ^b^	37.85 ± 1.40 ^c^	773(611,465,303)
6	Petunidin-3-*O*-(6-*O*-coumaryl)-glucoside	0.98 ±0.01 ^b^	0.89 ± 0.01 ^c^	1.03 ± 0.02 ^a^	8.55 ± 3.54 ^a^	3.37 ± 0.25 ^b^	5.85 ± 0.16 ^a, b^	787 (641, 625, 479, 317)
7	Petunidin-3-*O*-glucoside	3.43 ± 0.09 ^a^	2.55 ± 0.01 ^c^	3.26 ± 0.05 ^b^	122.93 ± 11.05 ^a^	76.95 ± 1.20 ^b^	76.12 ± 6.22 ^b^	641 (479, 317)
8	Petunidin-3-*O*-(6-*O*-acetyl)-glucoside	2.27 ± 0.08 ^a^	1.81 ± 0.01 ^b^	2.23 ± 0.02 ^a^	49.26 ± 6.73 ^a^	34.81 ± 1.35 ^b^	29.74 ± 1.56 ^b^	683 (641, 479, 317)
9	Malvidin-3-*O*-glucoside	74.53 ± 1.49 ^a^	58.13 ± 0.66 ^c^	68.54 ± 1.02 ^b^	553.20 ± 3.23 ^a^	415.00 ± 18.17 ^c^	485.76 ± 0.56 ^b^	655 (493, 331)
10	Malvidin-3-*O*-(6-*O*-acetyl)-glucoside	39.27 ± 0.70 ^a^	32.27 ± 1.12 ^c^	37.99 ± 0.52 ^b^	185.73 ± 24.57 ^a^	137.69 ± 4.26 ^c^	161.61 ± 5.49 ^b^	697 (655, 535, 493, 331)
11	Malvidin-3-*O*-(trans-6-*O*-coumaryl)-glucoside	5.11 ± 0.14 ^b^	3.70 ± 0.05 ^c^	5.28 ± 0.12 ^a^	52.00 ± 0.22 ^a^	30.12 ± 1.43 ^c^	46.38 ± 0.19 ^b^	801 (655, 639, 493, 331)
12	Malvidin-3-*O*-(cis-6-*O*-coumaryl)-glucoside	0.87 ± 0.01 ^a^	0.76 ± 0.02 ^b^	0.87 ± 0.01 ^a^	4.12 ± 0.20 ^a^	2.68 ± 0.04 ^b^	2.53 ± 0.14 ^b^	801 (655, 639, 493, 331)
13	Malvidin-3-*O*-glucoside-acetaldehyde	nd	nd	nd	38.13 ± 9.62 ^a^	25.66 ± 4.21 ^b^	9.29 ± 0.37 ^c^	697 (655, 535, 493, 331)
14	Malvidin-3-*O*-(6-*O*-acetyl)-glucoside-acetaldehyde	nd	nd	nd	10.22 ± 1.79 ^a^	6.73 ± 0.15 ^b^	7.16 ± 0.41 ^b^	517 (355)
15	Malvidin-3-*O*-(6-*O*-caffeoyl)-glucoside	nd	nd	nd	26.10 ± 5.30 ^a^	16.39 ± 0.66 ^b^	13.35 ± 0.85 ^b^	655 (493, 331)
16	Malvidin-3-*O*-(6-*O*-coumaryl)-glucoside-acetaldehyde	nd	nd	nd	3.36 ± 1.11 ^a^	2.89 ± 0.79 ^a, b^	1.80 ± 0.07 ^b^	639 (493, 331)
17	Malvidin-3-*O*-glucoside-4-vinylcatechol	nd	nd	nd	27.05 ± 2.38 ^a^	6.83 ± 0.30 ^c^	10.27 ± 0.76 ^b^	609(447 )
18	Peonidin-3-*O*-glucoside	2.43 ± 0.01 ^a^	1.88 ± 0.02^c^	2.39 ± 0.01 ^b^	226.81 ± 5.65 ^a^	157.19 ±0.32 ^c^	180.74 ± 4.57 ^b^	463 (301)
19	Peonidin-3-*O*-glucoside-pyruvic acid	nd	nd	nd	2.76 ± 0.91 ^a^	1.35 ± 0.60 ^b^	1.26 ± 0.06 ^b^	531 (369)
20	Peonidin-3-*O*-(6-*O*-acetyl)- glucoside	2.48 ± 0.03 ^a, b^	2.35 ± 0.06^b^	2.54 ± 0.15 ^a^	55.93 ± 5.15 ^a^	39.83 ± 0.51 ^c^	48.55 ± 3.14 ^b^	505 (343, 301)
21	Peonidin-3-*O*-(trans-6-*O*-coumaryl)-glucoside	0.82 ± 0.03 ^b^	0.69 ± 0.02^c^	0.91 ± 0.01 ^a^	18.98 ± 0.87 ^a^	11.81 ± 0.81 ^c^	15.06 ± 0.76 ^b^	771 (625, 609, 463, 301)
	Sum of anthocyanins	140.33 ± 2.45 ^a^	112.32 ± 1.77 ^c^	131.87 ± 1.93 ^b^	1660.86 ± 68.50 ^a^	1066.85 ± 20.09 ^c^	1168.39 ± 0.59 ^b^	
Flavonols (mg QE/L)								
22	Dihydroquercetin-3-hexoside	nd	nd	nd	9.71 ± 0.15 ^a^	6.23 ± 0.05 ^c^	6.41 ± 0.02 ^b^	465 (303)
23	Dihydroquercetin	nd	nd	nd	7.46 ± 0.10 ^a^	2.31 ± 0.14 ^b^	nd	303 (285, 125, 178)
24	Myricetin-3-glucoside	6.08 ± 0.04 ^a^	1.63 ± 0.01 ^b^	1.67 ± 0.01 ^b^	25.09 ± 0.31 ^a^	16.95 ± 0.13 ^c^	18.10 ± 0.09 ^b^	479 (317)
25	Quercetin-3-galactoside	1.49 ± 0.03 ^a^	0.94 ± 0.01 ^b^	0.93 ± 0.01 ^b^	10.48 ± 0.09 ^a^	1.23 ± 0.05 ^b^	1.35 ± 0.01 ^b^	463 (301)
26	Dihydroquercetin-3-rhamnoside	2.49 ± 0.04 ^a^	2.10 ± 0.03 ^b^	2.17 ± 0.02 ^b^	5.29 ± 0.08 ^b^	7.54 ± 0.06 ^a^	7.24 ± 0.05 ^a^	449 (303)
27	Quercetin-3-glucuronide	1.87 ± 0.04 ^a^	1.67 ± 0.02 ^b^	1.85 ± 0.01 ^a^	9.78 ± 0.16 ^a^	3.25 ± 0.09 ^b^	3.93 ± 0.02 ^b^	477 (301)
28	Quercetin-3-glucoside	2.38 ± 0.04 ^a^	2.36 ± 0.02 ^a^	2.50 ± 0.01 ^a^	9.42 ± 0.14 ^a^	6.31 ± 0.04 ^c^	7.43 ± 0.04 ^b^	463 (301)
29	Laricitrin-3-glucoside	1.64 ± 0.04 ^a^	1.48 ± 0.04 ^b^	1.58 ± 0.01 ^a^	6.47 ± 0.16 ^b^	7.02 ± 0.03 ^a^	7.27 ± 0.05 ^a^	493 (331)
30	Isorhamnetin-3-glucoside	1.57 ± 0.02 ^a^	0.97 ± 0.03 ^c^	1.08 ± 0.02 ^b^	12.83 ± 0.19 ^a^	8.53 ± 0.03 ^c^	9.57 ± 0.03 ^b^	477 (315)
31	Kaempferol-3-rutinoside	nd	nd	nd	15.18 ± 0.42 ^a^	nd	nd	593 (285)
32	Kaempferol-3-glucoside	2.32 ± 0.05 ^a^	1.32 ± 0.01 ^b^	1.41 ± 0.02 ^b^	nd	nd	nd	447 (285)
33	Syringetin-3-glucoside	1.28 ± 0.01 ^a^	1.03 ± 0.01 ^b^	1.08 ± 0.01 ^b^	10.22 ± 0.11 ^a^	7.21 ± 0.03 ^b^	7.63 ± 0.03 ^b^	507 (345)
34	Rutin	nd	nd	nd	14.10 ± 0.22 ^a^	5.94 ± 0.05 ^b^	6.33 ± 0.02 ^b^	301 (100)
	Sum of flavonols	21.12 ± 0.32 ^a^	13.5 ± 0.21 ^c^	14.26 ± 0.03 ^b^	136.01 ± 2.13 ^a^	72.52 ± 0.04 ^c^	75.26 ± 0.28 ^b^	
Flavan-3-ols (mg CE/L)								
35	Epigallocatechin	2.01 ± 0.03 ^a^	1.03 ± 0.01 ^b^	1.07 ± 0.01 ^b^	20.57 ± 0.29 ^a^	12.25 ± 0.12 ^b^	14.61 ± 0.02 ^b^	305 (179,241)
36	Catechin	32.33 ± 0.23 ^a^	23.24 ± 0.10 ^c^	26.64 ± 0.06 ^b^	61.60 ± 0.72 ^a^	39.56 ± 0.19 ^c^	44.91 ± 0.24 ^b^	289 (245, 205, 179)
37	Epicatechin	18.38 ± 0.14 ^a^	12.36 ± 0.04 ^c^	14.57 ± 0.05 ^b^	27.25 ± 0.33 ^a^	18.25 ± 0.09 ^c^	20.04 ± 0.08 ^b^	289 (245, 205, 179)
38	Proanthocyanidin dimer a	7.13 ± 0.09 ^a^	5.84 ± 0.04 ^c^	6.76 ± 0.02 ^b^	15.24 ± 0.20 ^a^	9.21 ± 0.17 ^c^	10.97 ± 0.06 ^b^	577 (425,289)
39	Proanthocyanidin dimer b	1.78 ± 0.02 ^a^	0.61 ± 0.01 ^c^	0.86 ± 0.01 ^b ^	1.57 ± 0.02 ^a^	1.03 ± 0.01 ^c^	1.70 ± 0.01 ^b^	577 (425,289)
40	Proanthocyanidin dimer c	8.19 ± 0.18 ^b^	8.31 ± 0.09^b^	8.63 ± 0.02 ^a^	25.21 ± 0.26 ^a^	18.26 ± 0.13 ^c^	19.66 ± 0.03 ^b^	577 (425,289)
41	Proanthocyanidin dimer d	8.34 ± 0.15 ^a^	5.61 ± 0.04^c^	6.47 ± 0.03 ^b^	1.90 ± 0.03 ^a^	0.61 ± 0.01 ^c^	0.98 ± 0.01 ^b^	577 (425,289)
42	Proanthocyanidin dimer e	4.70 ± 0.12 ^a^	1.82 ± 0.02^b^	1.94 ± 0.01 ^b^	30.78 ± 0.36 ^a^	17.13 ± 0.31 ^c^	20.49 ± 0.21 ^b^	577 (425,289)
43	Proanthocyanidin trimer a	7.03 ± 0.05 ^a^	6.42 ± 0.04^c^	6.06 ± 0.03 ^b^	7.72 ± 0.10 ^a^	5.61 ± 0.06 ^b^	5.97 ± 0.04 ^b^	865 (577,289 )
44	Proanthocyanidin trimer b	11.61 ± 0.03 ^a^	5.97 ± 0.07^c^	6.90 ± 0.07 ^b^	8.98 ± 0.21 ^a^	nd	nd	865 (577,289 )
45	Proanthocyanidin dimer-glucoside	11.01 ± 0.10 ^a^	5.76 ± 0.05^b^	5.75 ± 0.03 ^b^	20.69 ± 0.27 ^a^	12.53 ± 0.081 ^b^	12.83 ± 0.02 ^b^	729 (577,289)
	Sum of flavan-3-ols	112.52 ± 0.03 ^a^	76.97 ± 0.04 ^c^	85.65 ± 0.30 ^b^	221.52 ± 2.79 ^a^	134.44 ± 0.87 ^c^	152.17 ± 0.63 ^b^	
Stilbenes (mg RE/L)								
46	*trans*-Piceid	0.32 ± 0.01 ^a^	0.21 ± 0.01 ^b^	0.22 ± 0.01 ^b^	nd	nd	nd	389 (227)
47	Pallidol-3-glucoside	nd	nd	nd	1.20 ± 0.03 ^a^	nd	nd	453 (359,265)
	Sum of Stilbenes	0.32 ± 0.01 ^a^	0.21 ± 0.01 ^b^	0.22 ± 0.01 ^b^	1.20 ± 0.03 ^b^	—	—	
Hydroxybenzoic acids (mg GAE/L)								
48	Hexose ester of vanillic acid	1.53 ± 0.03 ^a^	nd	nd	0.26 ± 0.01 ^a^	nd	nd	329 (167)
	Sum of hydroxybenzoic acids	1.53 ± 0.03 ^a^	—	—	0.26 ± 0.01 ^a^	—	—	
Hydroxycinnamic acids (mg CAE/L)								
49	Caftaric acid	3.02 ± 0.04 ^b^	2.99 ± 0.01 ^b^	3.69 ± 0.01 ^a^	13.32 ± 0.18 ^a^	10.31 ± 0.01 ^b^	10.40 ± 0.04 ^b^	311 (179)
50	*p*-Coutaric acid	0.13 ± 0.01 ^a^	nd	0.04 ± 0.01 ^b^	6.17 ± 0.08 ^a^	4.59 ± 0.01 ^b^	4.77 ± 0.01 ^b^	295 (163)
51	*cis*-Fertaric acid	0.80 ± 0.02 ^a^	0.49 ± 0.01 ^b^	0.54 ± 0.01 ^b^	2.28 ± 0.03 ^a^	nd	nd	325 (193)
52	Hexose ester of *trans*-*p*-coumaric acid	nd	nd	nd	2.11 ± 0.02 ^a^	nd	nd	325 (163)
	Sum of hydroxycinnamic acids	3.95 ± 0.01 ^b^	3.48 ± 0.01 ^c^	4.26 ± 0.01 ^a^	23.88 ± 0.31 ^a^	14.9 ± 0.05 ^c^	15.17 ± 0.05 ^b^	
	Sum of phenolics	279.79 ± 3.33 ^a^	206.48 ± 5.40 ^c^	236.26 ± 7.34 ^b^	2043.72 ± 15.26 ^a^	1288.71 ± 11.89 ^c^	1410.99 ± 10.97 ^b^	

Results are mean ± standard deviation. Different letters (a,b,c) within the same column indicate significant difference at *p* < 0.05 by Duncan’s test. EBR: 0.40 mg/L 24-epibrassinolide. Brz: 1.00 mg/L brassinazole. Control: water. nd means no detected by 42.15% while the increase of CS wine was by 6.41%. One mg/L Brz treatment significantly decreased the total content of anthocyanins in both Yan73 and CS wines by 8.69 and 14.82%, respectively compared with the control.

Malvidin-3-*O*-glucoside, malvidin-3-*O*-(6-*O*-acetyl)-glucoside and peonidin-3-*O*-glucoside were major anthocyanins in Yan73 wine. They were accounted for 70.87% of the total anthocyanin concentration. Malvidin-3-*O*-glucoside and malvidin-3-*O*-(6-*O*-acetyl)-glucoside accounted for 80.78% of the total content in CS wine. The EBR treatment enhanced the major anthocyanins in the two wines. Malvidin-3-*O*-glucoside, the highest level anthocyanin in the two wines among the anthocyanins, showed a significant increase by 13.88% and 8.74% in the Yan73 and CS wine made from EBR treated grapes compared to the control, respectively. Malvidin-3-*O*-(6-*O*-acetyl)-glucoside was increased by 14.92% and 3.37% in Yan73 wine and CS wine, respectively. Peonidin-3-*O*-glucoside was increased by 25.49% in Yan73 wine. Moreover, malvidin-3-*O*-glucoside-acetaldehyde, cyanidin-3-*O*-(6-*O*-coumaryl)-glucoside and malvidin-3-*O*-glucoside-4-vinylcatechol in the Yan73 wine also increased by 551.16, 119.05, 310.44, 296.62 and 163.39%. Delphinidin-3-*O*-glucoside in the CS wine showed the highest increase by 42.29%. Brz treatment significantly decreased the malvidin-3-*O*-glucoside, malvidin-3-*O*-(6-*O*-acetyl)-glucoside and peonidin-3-*O*-glucoside in Yan73 wine by 14.57%, 14.80% and 13.03%, respectively. Malvidin-3-*O*-glucoside and malvidin-3-*O*-(6-*O*-acetyl)-glucoside of CS wine were decreased significantly by 15.19% and 15.06%, respectively.

#### 2.3.2. Flavonol Profiles

Twelve flavonols were identified in the Yan73 wine, while nine flavonols were found in the CS wine. The content of total flavonols in treated or untreated Yan73 wines was higher than that in CS wines. Compared with control, EBR increased the total flavonols content both in Yan73 wine and CS wine, by 80.72% and 48.12%, respectively. The CS wine treated by EBR showed a significant increase in the content of myricetin-3-glucoside, quercetin-3-galactoside, isorhamnetin-3-glucoside, kaempferol-3-glucoside, and syringetin-3-glucoside. On the other hand, the Yan73 wine treated by EBR showed a significant increase in the content of dihydroquercetin-3-hexoside, dihydroquercetin, myricetin-3-glucoside, quercetin-3-galactoside, quercetin-3-glucuronide, quercetin-3-glucoside, isorhamnetin-3-glucoside, kaempferol-3-rutinoside, syringetin-3-glucoside, and rutin compared to the control sample. Rutin was only found in Yan73 wines, and increased by EBR treatment.

#### 2.3.3. Flavan-3-ol Profiles

Compared to CS wines, Yan73 wines had high concentrations of flavan-3-ols. A significant (44.92%) increase in total content of flavan-3-ols of Yan73 wine treated by EBR was observed while the increase of CS wine was by 31.37%. Among the 11 detected flavan-3-ol compounds, three were monomers (epigallocatechin, catechin and epicatechin), five were dimers, two were trimers, and one was a dimer-glucoside. Except for proanthocyanidin dimer a and c in CS wine and proanthocyanidin dimer b in Yan73 wine, most of the flavan-3-ol compounds in treated wines were enhanced by EBR treatment. Interestingly, proanthocyanidin trimer b was only found in EBR-treated samples among Yan73 wines.

#### 2.3.4. Phenolic Acid Profiles

In wine, there are two groups of phenolic acids; hydroxybenzoic acids and hydroxycinnamic acids. One hydroxybenzoic acid, the hexose ester of vanillic acid, was found in the two cultivar wines after EBR treatment in this study. A total of four hydroxycinnamic acids, including caftaric acid, *p*-coutaric acid, *cis*-fertaric acid and hexose ester of *trans*-*p*-coumaric acid were detected in all wines. Caftaric acid was found to be the most predominant phenolic acids in the analyzed wine samples (Table 2). The increase of total hydroxycinnamic acids in Yan73 wine treated by EBR was observed by 57.42%.

#### 2.3.5. Stilbene Profiles

In this study, only pallidol-3-glucoside and *trans*-piceid were identified. *trans*-Piceid was detected in all CS wines, while pallidol-3-glucoside was detected only in Yan73 wine treated by EBR.

### 2.4. Antioxidant Capacity of Yan73 and CS Wines

The determined antioxidant capacities of Yan73 wine treated by EBR was significantly increased by 9.99% and 8.29%, in DPPH and HRSA assays, respectively (Table 3). The antioxidant capacities of CS wines treated by EBR were also significantly increased by 3.47% in the HRSA assay. For the two cultivar wines, neither EBR nor Brz treatment had any significant impact on the antioxidant activities relative to control in the ABTS method. The antioxidant activities of all samples treated by Brz were not significantly different from control, except a significant decrease in the CS wine was found by the HRSA assay.

**Table 3:** Antioxidant capacity of Yan73 and Cabernet Sauvignon wine produced with EBR-treated, Brz-treated, and untreated grape.

Variety	Treatments	DPPH (mmol/L)	ABTS (mmol/L)	HRSA (µmol/L)
Cabernet Sauvignon	EBR	4.94 ± 0.05 ^a^	7.83 ± 0.11 ^a^	12.53 ± 0.14 ^a^
	Brz	4.55 ± 0.12 ^b^	7.05 ± 0.53 ^b^	11.26 ± 0.19 ^c^
	Control	4.73 ± 0.07 ^a, b^	7.71 ± 0.26 ^a, b^	12.11 ± 0.15 ^b^
Yan 73	EBR	31.83 ± 1.41 ^a^	44.78 ± 0.88 ^a^	25.85 ± 0.51 ^a^
	Brz	27.97 ± 1.27 ^b^	38.84 ± 1.30 ^b^	22.42 ± 0.72 ^b^
	Control	28.94 ± 0.76 ^b^	42.50 ± 0.69 ^a, b^	23.87 ± 0.34 ^b^

Results are mean ± standard deviation. Different letters (a,b,c) within the same row indicate significant difference at *p* < 0.05 by Duncan’s test. EBR: 0.40 mg/L 24-epibrassinolide. Brz: 1.00 mg/L brassinazole. Control: water.

### 2.5. The Correlation between the Antioxidant Capacity and Phenolic Compounds

The correlation between the phenolic compounds and antioxidant capacity of Yan73 and CS wines was very significant. Table 4 shows that a linear correlation coefficients between the phenolic compounds and antioxidant capacity was measured by different methods. Pearson’s correlation analysis revealed high positive correlations between the TPC, TTC, TFC, TAC and antioxidant capacity. The correlations between the TFC and the antioxidant capacity were highest: 0.998, 0.999, 0.993 for DPPH, ABTS, HRSA, respectively (*p < 0.05*). These correlations suggested that the phenolic compounds were mainly responsible for the antioxidant capacity of Yan73 and CS wines, especially TFC.

**Table 4:** Linear correlation coefficients between the phenolic compounds and antioxidant capacity (panel A) obtained from the different methods (panel B).

		DPPH	ABTS	HRSA
**Panel A**				
**TPC**	Pearson correlation	0.991 ^**^	0.993 ^**^	0.995 ^**^
	Sig. (two-tailed)	0.009	0.007	0.005
**TTC**	Pearson correlation	0.993 ^**^	0.992 ^**^	0.997 ^**^
	Sig. (two-tailed)	0.007	0.008	0.003
**TFC**	Pearson correlation	0.998 ^**^	0.999 ^**^	0.993 ^**^
	Sig. (two-tailed)	0.002	0.001	0.007
**TAC**	Pearson correlation	0.959 ^*^	0.966^*^	0. 976 ^*^
	Sig. (two-tailed)	0.041	0.034	0.024
**Panel B**				
**DPPH**	Pearson correlation	1		
	Sig. (two-tailed)	0		
**ABTS**	Pearson correlation	0.999 ^**^	1	
	Sig. (two-tailed)	0.001	0	
**HRSA**	Pearson correlation	0.996 ^**^	0.996 ^**^	1
	Sig. (two-tailed)	0.004	0.004	0

* Correlation is significant at the 0.05 level (two-tailed); ** Correlation is significant at the 0.01 level (two-tailed).

### 2.6. Discussion

Brassinosteroids can greatly affect the developmental process during plant growth [24] and have been widely applied to increase the yield and protect against abiotic stress [25]. Brz, a brassinosteroid biosynthesis inhibitor, has been used frequently to confirm BR’s function in many plants, such as *Arabidopsis* [24,26], strawberry [18], cucumber [19] and grape [12,13]. Some information also suggests that BRs are the latest plant hormones implicated in the control of grape ripening [12]. Exogenous BR application could increase the phenolic contents and antioxidant capacity of grapes [13], while the impact of exogenous BR on phenolic profiles and antioxidant property of wines has not been documented.

In this study, it was found that EBR significantly increased the alcoholicity and dry extract of both Yan73 and Cabernet Sauvignon (CS) wines compared with the control. This could be explained by the fact that the application of EBR promoted the accumulation of carbohydrate in grape berries, which is consistent with the result that exogenous EBR enhanced the contents of reducing sugar and total soluble solids in berry juice of Yan73 and CS [13]. For acidity, the Yan73 grape berry had a higher level than some common wine grape varieties such as Merlot, Cabernet Franc and CS [27]. EBR treatment decreased significantly the total acid content of CS wine, but had no significant effect on that of Yan73 wine. It suggested that organic acids biosynthesis metabolism in CS grape berries may be inhibited by EBR and these reactions are variety-dependent.

Phenolics are critical components in relation to grape and wine quality. During the red winemaking process, phenolic compounds from the skins of red grapes transfer to the must during the fermentation and any maceration steps [28]. As for a monovarietal and young wine, the composition and content of phenolic compounds mainly depend on the variety. Total phenolic compounds (TPC, TTC, TFC and TAC) examined by spectrophotometric assays in Yan73 wines were significantly higher than these in CS wine. It is consistent with the previous study [10]. Besides no significant impact on TPC, TFC in Yan73 wine, EBR could increase the phenolic compounds in CS wine and other phenolic compounds in Yan73 wine significantly. However, Brz treatment decreased phenolic compounds of Yan73 and CS wines. The results were according with data determined by high performance liquid chromatography coupled with diode array detector and electrospray ionization mass spectrometry (HPLC-DAD/ESI-MS).

The phenolic profiles of Yan 73 and CS wines were assessed by HPLC-DAD/ESI-MS. For anthocyanins, the concentrations in the Yan73 wine were higher than those in the CS wine. They also were found to be the most predominant phenolic compound in all samples (Table 2). The anthocyanins contained within the grape berries are extracted into wines, mostly from the berry skins. Xi *et al.* [13] found that EBR treatment increased the accumulation of anthocyanins in the Yan73 and CS grape skins. Moreover, Ma *et al.* [11] also reported that EBR treatment could significantly enhance the Yan73 grape berries coloration. In our study, there is a significant change by EBR or Brz treatment to the five major anthocyanins in Yan73 and CS wines. Flavonols, which seem to be responsible for bitterness and color, contribute to the color stabilization of red wines by reinforcing the pigmentation due to anthocyanins. It was easily found that the flavonols in wines produced by EBR-treated grapes had a higher level (Table 2). In addition, the contents of characteristic flavonols (dihydroquercetin-3-hexoside and dihydroquercetin) of Yan 73 wines were increased significantly. Considering that dihydroflavonols (flavanonols) are precursors of flavonols [29], the presence of dihydroflavonols and the low concentration of flavonol could be related to a lower activity for flavonol synthase (FLS) in grapes under EBR treatment compared with those under Brz-treated and untreated grapes. The last group of flavonoids is the flavan-3-ols, which are found in the solid parts of the berry (seed, skin, and stem) in monomeric, oligomeric, or polymeric forms; the latter 2 forms are also called proanthocyanidins or condensed tannins. Both the total tannin content (TTC) and flavan-3-ol profiles showed that EBR treatment could increase the levels of flavan-3-ols in wines. In grapes, the biosynthesis of flavanol monomers involves the leucoanthocyanidin reductase (LAR) and anthocyanidin reductase (ANR). LAR is responsible for the synthesis of (+)-catechin. ANR catalyzes the conversion of some grape anthocyanidins (specifically delphinidin and cyanidin) to (+)-catechin and (−)-epicatechin. EBR treatment could regulate *VvLAR* and *VvANR* expression to promote flavan-3-ols accumulation (unpublished). As seen in the above data, all of flavonoids were increased by EBR treatment. Flavonoids are the products of the secondary metabolism and shared a common phenylpropanoid synthetic pathway [4]. They are the down-stream products of this pathway. The activity of phenylalanine ammonialyase (PAL) and flavonoid 3-O-glucosyltransferase (UFGT) that catalysis the phenylpropanoid synthetic pathway were enhanced by EBR [13].

Since stilbenes are phytoalexins and their existence in grapes is directly related to environmental stress, such as *Botrytis* infections and UV-irradiation [30], healthy plants contain small amounts. The increase of stilbenes in EBR-treated wines were significant, and suggested that this plant hormone may be essential for stress response [16,17].

In summary, the phenolic compounds in the wines made from the grapes treated by EBR have higher levels, and even some individual phenolics only detected in EBR-treated wines, such as kaempferol-3-rutinoside, proanthocyanidin trimer b, pallidol-3-glucoside, hexose ester of vanillic acid, hexose ester of *trans*-*p*-coumaric acid. Nevertheless, there were different levels of promotion of EBR in two variety wines. This may be due to variation in the genetic make-up of the two cultivars, thus leading to different EBR responses. The vineyards used for these trials had similar weather, viticultural practices, and soil conditions with the same winemaking technology. Minor differences in the environmental factors, cultivation management and winemaking process were less likely to be the factors that caused different EBR responses in two cultivars.

Since there is no standardized method for the determination of antioxidant capacity, three methods (DPPH, ABTS and HRSA) based on different reaction mechanisms were used to carry out the antioxidant capacity measurements [31]. Phenolics are directly responsible for the antioxidant capacity in young wines and contribute to antioxidant activity [32]. In present study, the increase or decrease in the antioxidant capacities determined by three different assays was consistent with the higher or lower level of total phenolics, tannins, flavonoids and anthocyanins in wines treated by EBR or Brz, respectively. Compared with our previous study [10], both ABA and EBR could increase TPC, TTC, TFC, and TAC of CS and Yan73 wines. However, the increase of antioxidant capacity of the two cultivar wines for ABA treatment was higher than that with EBR treatment. It is explained that the increase of TPC, TTC, TFC, and TAC of CS and Yan73 wines promoted by ABA were higher than the influence of EBR treatment. It also suggested that the antioxidant capacity was determined by the level of phenolic compounds in red wines. Flavan-3-ols are the most important compounds that contribute to the red wine antioxidant properties [33,34]. The significantly higher values obtained in red wines for antioxidant capacity was explained by the higher levels of polyphenols in red wines [32,35]. Our findings also showed that the flavonoids are the major compounds contributing to total phenolics and antioxidant capacity. Other non-flavonoids phenolics had less contribution to antioxidant capacity. It is consistent with Katalinic’s [36] conclusion that a linear correlation exists between the antioxidant capacity and flavonoids fraction but not free anthocyanidins fraction.

As a BRs biosynthesis inhibitor, Brz delayed Cabernet Sauvignon grape [12] and Akihime strawberry fruit ripening [18] and decreased phenolic compounds and antioxidant capacity of Yan73 and CS wines in this study. Surprisingly, there were significant increase of some compounds in the two cultivar wines compared with the control, including delphinidin-3-*O*-glucoside and cyanidin-3-*O*-glucoside. In grape berries, *O*-methyltransferases (OMTs) catalyze the methoxylation of 3'-OH of cyanidin-3-*O*-glucoside to generate peonidin-3-*O*-glucoside, and the methoxylation of 3'-OH of delphinidin-3-*O*-glucoside to form petunidin-3-*O*-glucoside [6]. It suggested that the biosynthesis and/or activity of OMTs may be inhibited by endogenous BRs.

## 3. Experimental Section

### 3.1. Reagents and Equipment

Deionized water (<18 MΩ resistance) was obtained from a Milli-Q Element water purification system (Millipore, Boston, MA, USA). Methanol and glacial acetic acid (≥99%, HPLC grade) were purchased from Fisher (Fairlawn, NJ, USA). 24-Epibrassinolide (≥99%), brassinazole (≥99%) and polyvinylpyrrolidone (PVP-30, >98%) were purchased from Sigma Chemical Co. (St. Louis, MO, USA). 6-Hydroxy-2,5,7,8-tetramethylchroman-2-carboxylic acid (Trolox, >98%), 1,1-Diphenyl-2-picrylhydrazyl radical (DPPH, >98%), 2,2-azinobis(3-ethylbenzothiazoline-6-sulphonic acid) diammonium salt (>98%, ABTS) and (Φ)-catechin (>99%) were also supplied by Sigma Chemical Co. All other reagents used were of analytical grade (all over 97%) and purchased from Xi’an Chemical Factory (Xi’an, China). A Shimadzu UV-1700 UV-visible spectrophotometer (Kyoto, Japan) was employed to detect the absorbance.

### 3.2. Experimental Design and EBR Treatments of Grape Berries

The experimental vineyard was located at Jingyang County, Shaanxi Province, China. Two cultivars of *V.vinifera* grape, Yan73 and Cabernet Sauvignon, were the experimental materials. Yan73 was obtained by crossing Muscat Hamburg (*Vitis vinifera*) × Alicante Bouschet (*Vitis vinifera*) in 1966. It contains red and purple pigments in the skin and pulp. Cabernet Sauvignon is one of the most commonly used wine grape varieties. In the study, both of the own-rooted Yan73 and Cabernet Sauvignon grapevines had similar growth conditions. The vines were spaced 0.8 m in row and 2.5 m between the rows which were oriented in South-North direction. They were trained on a vertical shoot-positioning system with a pair of wires. The shoots were trimmed twice manually, between bloom and version, to a height of approximately 1.0 m.

Sixty grapevines of each variety were selected and assigned to receive one of the three treatments by spraying deionized water (control), 0.40 mg/L 24-epibrassinolide (EBR), 1.00 mg/L brassinazole (Brz). The concentration of EBR and Brz chosen for treatment was based on our previous experiments on Yan73 and CS [11]. The stock solutions of EBR were prepared by dissolving EBR in 1 mL of ethanol (98%). The control stock solution was 1 mL 98% ethanol without adding EBR. Each of them was mixed with 1 mL of Tween 80 and diluted to 1 L using sterilized water. Ten ml of each concentration of EBR solution was applied per cluster by spraying on all surface area of the berries on that cluster. The application dates were July 2nd for Yan73 and July 9th for Cabernet Sauvignon. Each treatment had three independent replicates. Each replicate consisted of five grapevines. All the clusters of each tree, including 15 clusters, were sprayed with the treated solution or water. Two hundred berries were randomly sampled from each treatment after they reached the commercial maturity. 

### 3.3. Winemaking

Pre-fermentation treatments and winemaking were done as described by Li *et al.* [37]. Grapes were destemmed and crushed on an experimental destemmer-crusher and then transferred to stainless steel containers. Forty liters of each treatment wine were produced in three replications. Sixty mg/L of SO_2_ and 30 mg/L of pectinase (Lallzyme Ex) were added to the must, respectively, with adding 20 g/L of dried active yeast (*Saccharomyces cerevisiae* RC 212, Lallemand, Danstar Ferment AG, Massagno, Switzerland) according to its commercial specifications. Maceration was carried out at the same time as the fermentation, which took place over an 8-day period at 25–28 °C. After the fermentation, wines were decanted to another tank, stabilized for 6 months at 4 °C, and then bottled. Phenolic compounds were analyzed for each wine immediately after bottling.

### 3.4. Determination of the Physicochemical Parameters

Alcohol content, reducing sugar, total acidity, volatile acidity and dry extract of these wines at the moment of bottling were analyzed according with the methods proposed by OIV [23].

### 3.5. Determination of Total Phenolic, Tannin, Flavonoid and Anthocyanin Content

Total phenolic content (TPC) and total tannin content (TTC) were determined according to the bovine serum albumin (BSA) precipitation method [38]. All buffer solutions were prepared before the experiment. Buffer A was a washing buffer of 200 mM acetic acid and 170 mM sodium chloride, pH adjusted to 4.9 with sodium hydroxide. Buffer B was a model wine (5.0 g/L potassium bitartrate and 12% (v/v) ethanol, pH adjusted to 3.3 with HCl. Buffer C was a resuspension buffer consisting 5% (v/v) triethanolamine and 5% (w/v) sodium dodecyl sulphate, pH adjusted to 9.4 with HCl. Ferric chloride reagent was 0.01 M HCl and 10 mM ferric chloride.

For TTC determination, a protein solution for tannin precipitation was prepared by dissolving bovine serum albumin (BSA) in buffer A, to give a final protein concentration of 1.0 mg/mL. The skin extract was diluted with buffer B, 1.0 mL of the protein solution and 500 μL diluted extract sample a 1.5 mL microfuge tube. After being incubated for 15 min with slow agitation at room temperature, the mixture was centrifuged at 14,000 g for 5 min at 4 °C. After the supernatant was poured out, the residue was washed with buffer A three times and then resolublized in 875 μL of buffer C. Then, 125 μL of ferric chloride reagent was added and shaken for 10 min. The absorbance of the solution was read at 510 nm for tannin background A_510_. Then, 125 μL of ferric chloride reagent was added and shaken for 10 min. The solution were read at 510 nm for tannin final A_510_. Buffer C was used as a blank and read at 510 nm for tannin initial A_510_.

For TPC, 20.0 mL of wine sample and 855 mL of buffer C were mixed. After being incubated for 10 min, the mixture was read at 510 nm (total phenolics background A_510_). Next, 855 mL of ferric chloride regent was added into the reaction system. The absorbance was read at 510 nm (total phenolics final A_510_). The absorbance for TTC is:

[(tannin final A_510_) − (tannin initial A_510_)] − (tannin background A_510_) × 0.875.

The absorbance for TPC is:

[(total phenolics final A_510_) − (tannin initial A_510_)] − (total phenolics background A_510_) × 0.875.

Phenolics or tannin content was calculated using a calibration curve of (+)-catechin standard and expressed in catechin equivalents (CE). Total flavonoid content (TFC) was determined according to the method of Jia *et al.* [39] with minor modifications. In a centrifuge tube, 1.0 mL of grape extract was mixed with 4.4 mL of ethanol (70%) solution, 0.3 mL of NaNO_2_ (0.5 M) and 0.3 mL of AlCl_3_ (0.3 M) in sequence. After 5 min, 4.0 mL of NaOH (1.0 M) was added to the reaction system. The absorbance was measured against the blank at 510 nm. Results were expressed as rutin equivalents (RE).

Total anthocyanin content (TAC) was estimated using the pH differential method [5]. Each wine extract was diluted with buffers at pH 1.0 and 4.5 to attain the same dilution. The absorbance was measured at 520 and 700 nm in both pH 1.0 and 4.5 buffers. The TAC (expressed in terms of cyanidin-3-glucoside) was calculated using the following formula:

TAC = A × DF × MW × 1000/(ε × C)

A = (A_520_ − A_700_)_pH1.0_ − (A_520_ − A_700_)_pH4.5_

where MW is the molecular weight of cyanidin-3-glucoside (449 g/mol), DF is the dilution factor, ε is the molar extinction coefficient of cyanidin-3-glucoside (29,600) and C is the concentration of extracted volume.

### 3.6. HPLC-DAD/ESI-MS Analysis of Phenolic Profiles

The chromatographic analyses of anthocyanins were performed using an Agilent 1100 series LC-MSD trap VL (Agilent Corporation, Santa Clara, CA, USA) equipped with a G1379A degasser, a G1311A quaternary pump, a G1313A ALS autosampler, a G1315B photodiode array detector and a reversed phase column (Kromasil C18, 250 × 4.6 mm, 5 μm). The mobile phase was: 6% (v/v) acetonitrile containing 2% (v/v) formic acid as solvent A, and 54% (v/v) acetonitrile containing 2% (v/v) formic acid as solvent B. The elution profile had the following proportions (v/v) of solvent B: 0.00–1.00 min, 10%; 1.00–18.00 min, 10%–25%; 18.00–20.00 min, 25%; 20.00–30.00 min, 25%–40%; 30.00–35.00 min, 40%–70%; 35.00–40.00 min, 70%–100%; 40.00–45.00 min, 100%–10%. The column was held at 50 °C and was flushed at a flow rate of 1.0 mL/min. The injection volume was 30 µL. Diode array detection was performed from 200 to 900 nm and quantification was carried out by peak area measurements at 525 nm. MS conditions were as follows: Electrospray ionization (ESI) interface, positive ion model, 35 psi, 10 mL/min dry gas flow rate, 325 °C dry gas temperature, and scans between *m/z* 100 and 1,000 [40].

Non-anthocyanins analyses were performed using an Agilent 1200 series LC-MSD trap XCT (Agilent Corporation) equipped with a G1322A degasser, a G1312B binary pump, a G1367C HiP-ALS autosampler, a G1316B TCC (thermostated column compartment), a G1314C VWD (variable wavelength detector) and a reversed phase column (Zorbax SB-C18, 3 × 50 mm, 1.8 μm). The mobile phase A was a water solution with 1% acetic acid and the mobile phase B was an acetonitrile solution with 1% acetic acid. The elution profile had the following proportions (v/v) of solvent B: 0.00–5.00min, 5%–8%; 5.00–7.00 min, 8%–12%; 7.00–12.00 min, 12%–18%; 12.00–17.00 min, 18%–22%; 17.00–19.00 min, 22%–35%; 19.00–21.00 min, 35%–100%; 21.00–25.00 min, 100%; 25.00–27.00 min, 100%–5%;. The column was held at 25 °C and was flushed at a flow rate of 1.0 mL/min. The injection volume was 2 µL and analyses were detected at 280 nm. MS analysis was used ESI, negative ion model, 35 psi nebulizer pressure, 10 mL/min dry gas flow rate, 325 °C dry gas temperature, and scans between *m/z* 100 and 1000 [40]. All phenolic compounds was identified by comparison of their order of elution and retention time with those of standards and the weight of molecular ion and the fragment ion compared with standards and references [41]. Quantitative determinations were made by using the external standard method with the commercial standards. The calibration curves were obtained by injection of standard solutions under the same conditions as for the samples analyzed, over the range of concentrations observed. Anthocyanins, flavonols, flavan-3-ols, hydroxybenzoic acids, hydroxycinnamic acids and stilbenes were respectively expressed as micrograms of malvidin-3-O-glucoside (ME), quercetin equivalence (QE), catechin equivalence (CE), gallic acid equivalence (GAE), caffeic acid equivalence (CAE), and resveratrol equivalence (RE)/L of wine.

### 3.7. Determination of Antioxidant Capacity

DPPH free radical-scavenging capacity was estimated using the method of Brand-Williams *et al*. [42]. One-tenth mL of wine (diluted at 1:20) was added to 3.9 mL of a 6 × 10^−5^ M solution of DPPH in methanol. A control sample, containing the same volume of solvent stead of extract, was used to measure the maximum DPPH absorbance. After the reaction was allowed to take place in dark for 30 min, the absorbance at 517 nm was recorded to determine the concentration of remained DPPH. Results were expressed as micromoles of Trolox equivalents per liter of wine.

ABTS free radical-scavenging capacity was based on the slightly modified method of Re *et al*. [43]. ABTS radical cation (ABTS^+^) was produced by reacting 7 mM ABTS solution with 2.45 mM potassium persulphate aqueous solution and allowing the mixture to stand in the dark at room temperature for 12–16 h before use. The ABTS^+^ solution was diluted with ethanol. After addition of 0.1 mL of wine (diluted at 1:20) to 3.9 ml of diluted ABTS^+^ solution, the solution was measured for its absorbance at 732 nm after exactly 8 min. Results were expressed as micromoles of Trolox equivalents per liter of wine.

Hydroxyl radical-scavenging activity (HRSA) was estimated using the method described by Meng *et al.* [5] with a slight modification. Briefly, 3.0 mL of FeSO_4_ (2 mM), 3.0 mL of salicylic acid (6 mM) and 1.0 mL of wine were sequentially mixed with 4.0 mL of distilled water. Then, 3.0 mL of H_2_O_2_ (1 mM) was added to this mixture and reacted for 30 min at 37 °C. The absorbance of the colored product was measured at 593 nm. The results were expressed as micromoles of trolox equivalents per liter of wine.

### 3.8. Statistical Analysis

Data were reported as mean standard deviation (SD) values of triplicate experiments and analyzed using SPSS 17.0 for Windows (SPSS, Inc., Chicago, IL, USA). One-way analysis of variance (ANOVA) and Duncan’s multiple range tests were used to determine the significance of the difference among samples, with a significance level of 0.05. A two-tailed Pearson’s correlation test was conducted to determine the correlations among means. 

## 4. Conclusion

Exogenous BR application enhanced the phenolic compounds of CS of Yan73 wines, whereas Brz inhibited TPC, TFC, and TAC of CS wine and TTC, TFC, and TAC of Yan73 wine. Individual phenolic compounds in the wines were changed by different levels. Generally, the wine made from the grapes treated by EBR have higher levels of phenolic compounds and antioxidant capacity and greater health benefits. EBR treatment applied at critical stages of grape development to grape berries is a promising tool for enhancing the phenolic compounds in the wine.
